# Investigating the Roles of the Visual and Parietal Cortex in Representing Content versus Context in Visual Working Memory

**DOI:** 10.1523/ENEURO.0270-20.2024

**Published:** 2024-02-06

**Authors:** Chunyue Teng, Bradley R. Postle

**Affiliations:** ^1^University of Wisconsin–Madison, Madison 53719, Wisconsin; ^2^Lawrence University, Appleton 54911, Wisconsin

**Keywords:** attention, working memory

## Abstract

Content-to-context binding is crucial for working memory performance. Using a dual-serial retrocueing (DSR) task on oriented gratings, Yu et al. (2020) found that content (orientation) of both prioritized and unprioritized memory items (PMI; UMI) was represented simultaneously in visual cortex, while their context (location) was represented in intraparietal sulcus (IPS), with a priority-based remapping of the representation of content and context of the UMI in each region, respectively. This registered report acquired fMRI of 24 healthy adults while they performed a DSR task with location as the to-be-reported content and orientation as the task-relevant context. We contrasted three accounts: domain-dependent, the engagement of visual and parietal regions depends on the feature domain (orientation vs location); functional, the engagement of these regions depends on their function (content vs context); and hybrid—a combination of the domain-dependent account and the additional stipulation that IPS encodes context regardless of domain. Delay-period activity in early visual cortex conformed most closely with functional predictions: robust priority-sensitive representation of stimulus location (content), but no evidence for the active representation of stimulus orientation (context). Delay-period activity in IPS, in contrast, conformed most closely to predictions of the hybrid account: active representation of content (location) and of prioritized context (orientation). Exploratory analyses further supported the hybrid account of IPS, revealing univariate sensitivity to variation in both content and context load, the latter in a manner that predicted individual differences in behavior. The representation of visual information in working memory is highly dependent on behavioral context.

## Significance Statement

The binding between the content of working memory and the context in which that information was encountered is of critical importance for guiding behavior with remembered information. When attention is shifted away from a stimulus held in working memory, the representation of its content and of its context are transformed, but in different brain areas. This preregistered report is designed to understand the principal factor underlying this dissociation: is it that different regions are specialized for representing different domains of content (e.g., an item's orientation vs its location); that different regions are specialized for different working memory functions (i.e., representing content vs context); or some hybrid combination of these two? The results will provide important insights into the mechanisms that support visual working memory.

## Introduction

The retention of information in visual working memory entails the binding of item-related information—the to-be-remembered content—with its trial-unique context (e.g., spatial location or the ordinal position). Furthermore, it has been proposed that the strength of this content-to-context binding may be an important determinant of the precision of a memory representation (Oberauer and Lin, 2017). Although several studies have assessed the neural representation of to-be-remembered stimulus features (Harrison and Tong, 2009; Riggall and Postle, 2012; Emrich et al., 2013; Sprague and Serences, 2013), the representation of context has received less attention (cf. Foster et al., 2017; Gosseries et al., 2018). The current study is designed to assess whether the brain represents the same information differently when it serves as working memory context, rather than content.

In a recent experiment, Yu et al. (2020) assessed the representation of content and of context in visual working memory with functional magnetic resonance imaging (fMRI). In a dual-serial retrocueing (DSR) task, two sample orientation gratings were presented sequentially in one of nine possible locations, and then an ordinal retrocue (“1” or “2”) indicated the sample whose orientation would need to be reported for the first impending recall. After the first recall, subjects perform the second recall based on a second retrocue. Prior to the first retrocue, using multivariate inverted encoding modeling (IEM), the orientation of both sample stimuli could be reconstructed in early visual cortex and their locations in early visual cortex and intraparietal sulcus (IPS). The first retrocue then designated the cued item the “prioritized memory item” (PMI) and the uncued item the “unprioritized memory item” (UMI), and the transition to UMI triggered distinctive changes in its representational format. In early visual cortex, the reconstruction of the UMI's orientation became opposite of its reconstruction as a PMI, whereas in IPS, the reconstruction of the UMI's location shifted to become opposite of its reconstruction as a PMI. Importantly, these reported priority-based transformations were characterized as examples of remapping between the stimulus values and the neural patterns, not of recoding (Yu et al., 2020), because they were reconstructed with IEMs trained on the PMI.

This phenomenon of “priority-based remapping” has also been observed in EEG data from Wan et al. (2020) and in computational simulations of Wan et al. (2022) of a two-back working memory task. In the present paper, we will use it as a tool to test competing models of the neural representation of content versus context in visual working memory. The results from Yu et al. (2020) are equally consistent with at least three interpretations. One is that neural loci of the representation of an item's content and of its context are domain dependent. By this account, early visual cortex carries a privileged role in representing orientation, and IPS carries a privileged role in representing retinotopic location. An alternative interpretation, however, is a functional account: Early visual cortex may be preferentially involved in representing an item's content, and IPS in representing an item's context. This alternative view would be consistent with evidence for IPS sensitivity to context binding demands when task-critical context is ordinal position, and location does not vary (Gosseries et al., 2018). A third possibility is a hybrid view that ascribes to the domain-dependent processing of orientation in early visual cortex and of location in IPS, but that also posits an important functional role for IPS in representing the context of information, regardless of domain. This view would be consistent with findings of retinotopic maps in parietal cortex (Sereno et al., 2001; Silver et al., 2007) and with evidence for IPS's involvement in context binding with ordinal context (Gosseries et al., 2018).

**Figure 1.**
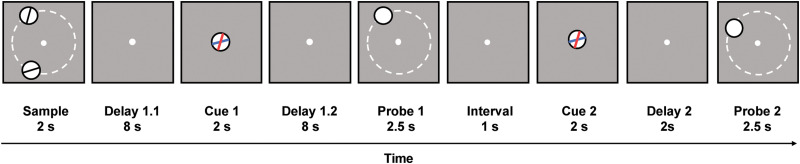
Procedures of the experiment. Subjects performed a DSR task on location. Two sample stimuli were presented at nine possible locations with six possible orientations and the task was to memorize the spatial locations of both. The white dotted circles are for illustrative purposes and were present during the actual experiment. The distances in orientations and locations between the two stimuli were fully counterbalanced, such that they matched on 1/9th and 1/6th of trials, respectively. After Delay 1.1, an orientation cue (superimposed red and blue oriented bars inside a central disk) appeared with the red bar (or blue bar, counterbalanced between subjects) indicating the sample whose location was to be reported during Probe 1. Probe 1 consisted of a filled white disk that appeared in a location either completely matching or slightly mismatching the location that the cued sample had occupied, and subjects made a same/different judgment on its location. Subsequently, a second orientation cue appeared after an interval of 1 s, and subjects responded during Probe 2 based on the red oriented bar. Each trial was separated by an ITI jittered between 6, 8, and 10 s.

The purpose of the present study is to compare among the three interpretations of the results from Yu et al. (2020), by switching the roles played by stimulus orientation and location. Subjects will perform a DSR task in which the two sample stimuli are each distinguished by their location and their orientation, but the subject's task is explicitly to recall stimulus location (Fig. 1). The item to be recalled for each memory probe will be retrocued by its orientation. Thus, stimulus location will serve as the content and stimulus orientation as the context. Priority-based remapping during the delay period following the first retrocue will be operationalized by a negative slope of the reconstruction of a stimulus dimension of the UMI during the final TR of the post-cue delay, with an IEM trained on that same TR during trial epochs when that item was the PMI.

## Materials and Methods

### Preregistered hypotheses

**Figure 2.**
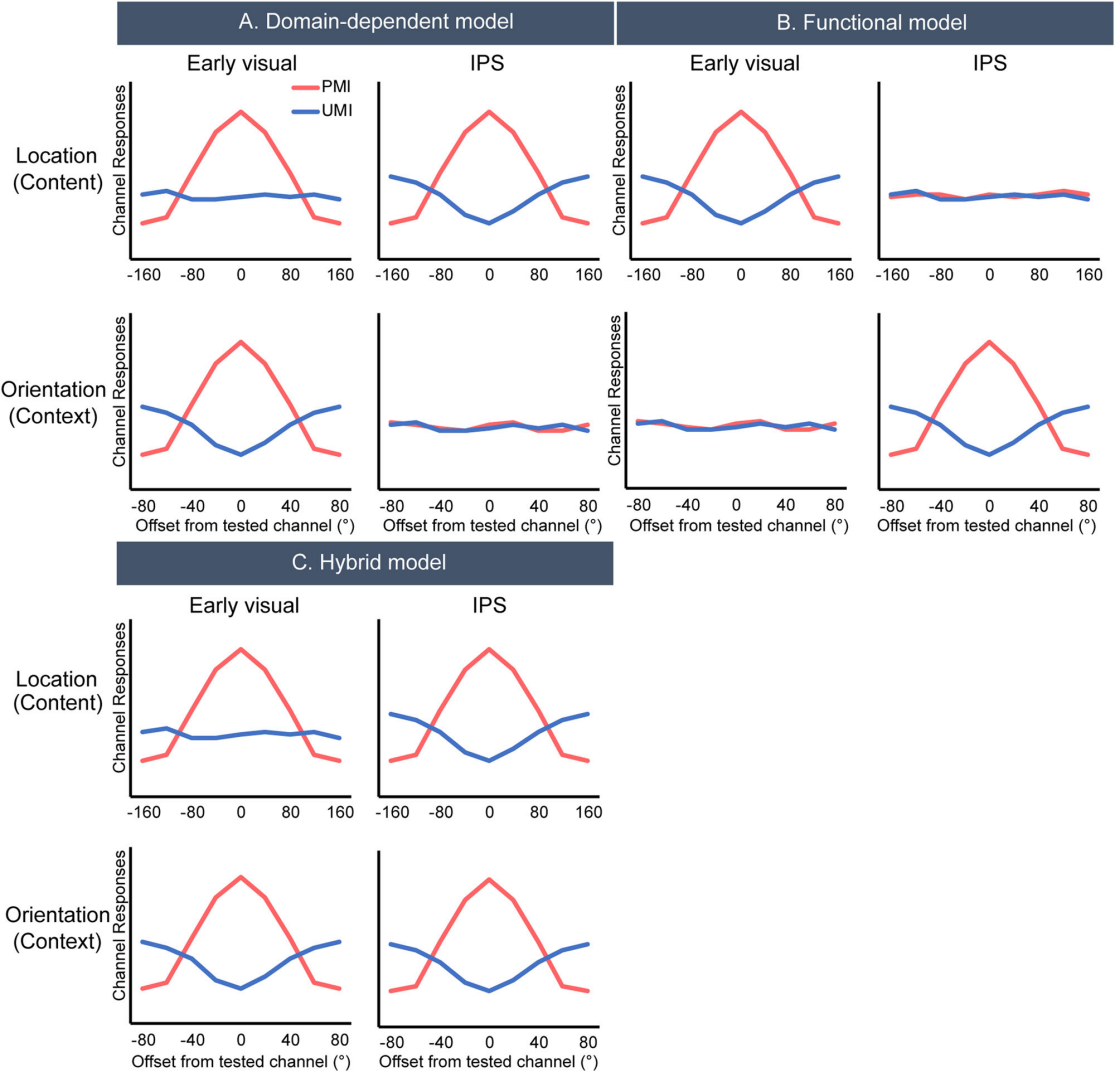
Predictions of location and orientation reconstructions according to the three different models. We consider the opposite patterns between PMI and UMI as evidence for priority-based remapping. ***A***, Domain-dependent model predicts priority-based remapping for orientation in early visual cortex and location in IPS. ***B***, Functional model predicts priority-based remapping for content in early visual cortex and context in IPS. ***C***, Hybrid model predicts domain-dependent priority-based remapping for orientation in early visual cortex and location in IPS, as well as context (orientation) being encoded in IPS.

In this section, for each of the following results that our study is designed to generate, we specify what each of the three models predicts for the IEM reconstruction of stimulus information in two functionally defined regions of interest (ROI; these predictions are illustrated in Fig. 2).

#### Content of PMI in early visual cortex

All three models assume that the reconstruction of the location of the PMI with a PMI-trained model will have a significantly positive slope, a replication of Yu et al. (2020).

#### Context of PMI in early visual cortex

##### Domain-dependent

The reconstruction of the orientation of the PMI with a PMI-trained model will have a significantly positive slope.

##### Functional

The reconstruction of the orientation of the PMI with a PMI-trained model will not differ from 0.

##### Hybrid

The reconstruction of the orientation of the PMI with a PMI-trained model will have a significantly positive slope.

#### Content of UMI in early visual cortex

##### Domain-dependent

The reconstruction of the location of the UMI with a PMI-trained model will not differ from 0.

##### Functional

The reconstruction of the location of the UMI with a PMI-trained model will have a significantly negative slope.

##### Hybrid

The reconstruction of the location of the UMI with a PMI-trained model will not differ from 0.

#### Context of UMI in early visual cortex

##### Domain-dependent

The reconstruction of the orientation of the UMI with a PMI-trained model will have a significantly negative slope.

##### Functional

The reconstruction of the orientation of the UMI with a PMI-trained model will not differ from 0.

##### Hybrid

The reconstruction of the orientation of the UMI with a PMI-trained model will have a significantly negative slope.

#### Content of PMI in IPS

##### Domain-dependent

The reconstruction of the location of the PMI with a PMI-trained model will have a significantly positive slope.

##### Functional

The reconstruction of the location of the PMI with a PMI-trained model will not differ from 0.

##### Hybrid

The reconstruction of the location of the PMI with a PMI-trained model will have a significantly positive slope.

#### Context of PMI in IPS

##### Domain-dependent

The reconstruction of the orientation of the PMI with a PMI-trained model will not differ from 0.

##### Functional

The reconstruction of the orientation of the PMI with a PMI-trained model will have a significantly positive slope.

##### Hybrid

The reconstruction of the orientation of the PMI with a PMI-trained model will have a significantly positive slope.

#### Content of UMI in IPS

##### Domain-dependent

The reconstruction of the location of the UMI with a PMI-trained model will have a significantly negative slope.

##### Functional

The reconstruction of the location of the UMI with a PMI-trained model will not differ from 0.

##### Hybrid

The reconstruction of the location of the UMI with a PMI-trained model will have a significantly negative slope.

#### Context of UMI IPS

##### Domain-dependent

The reconstruction of the orientation of the UMI with a PMI-trained model will not differ from 0.

##### Functional

The reconstruction of the orientation of the UMI with a PMI-trained model will have a significantly negative slope.

##### Hybrid

The reconstruction of the orientation of the UMI with a PMI-trained model will have a significantly negative slope.

### Subjects

Estimated effect sizes for this preregistered study were based on datasets from Yu et al. (2020) that used data collection and analysis methods similar to what we used for this preregistered study. Power analyses indicated we needed data from 24 subjects to achieve 90% power to detect the smallest of the predicted significant effects (significantly negative slope for the reconstruction of orientation of the UMI in early visual cortex, Cohen's *d* of 0.62) with a one-tailed alpha of 0.05.

Twenty-four subjects (12 male and 12 female; *M* of age = 22.4; SE = 4.7) were recruited at the University of Wisconsin–Madison with the following inclusion criteria: being 18–35 years in age; having normal or corrected-to-normal vision; reporting no history of neurological disease, seizures, or fainting; reporting no history of using of psychotropic drugs nor of chronic alcohol consumption; and having no contraindications for MRI scanning. Informed consent was obtained following procedures approved by the University of Wisconsin–Madison Health Sciences Institutional Review Board.

### Stimuli and procedure

The stimuli used in the task were generated with MATLAB (MathWorks) and the Psychtoolbox-3 extensions and presented with a 60 Hz Avotec Silent Vision 6011 projector (Brainard, 1997; Pelli, 1997). Subjects viewed the stimuli through a coil-mounted mirror, with a viewing distance of 68.58 cm and the screen width of 33.02 cm. The stimuli during the sample period were two oriented bars colored black (length, 5°; width, 0.08°; presented inside a white disk of radius of 2.5°). The orientation of the two bars was selected randomly, with replacement, from a fixed set of values (15°, 45°, 75°, 105°, 135°, or 165°; ∼54 instances of each). The locations of the disks were selected randomly, with replacement, from a fixed set of nine values of polar angle (20°, 60°, 100°, 140°, 180°, 220°, 260°, 300°, 340°; ∼36 instances of each), each centered on an imaginary circle with a radius of 8° from central fixation. In order to cover the 360° space and avoid verbal encoding, on each trial, a jitter between 0° and 10° was added to both sample locations with the same value. There could be six possible distances between the PMI and UMI in orientation space, −60°, −30°, 0°, 30°, 60°, and 90°, and nine possible distances between the PMI and UMI along the imagery circle: −160°, −120°, −80°, −40°, 0°, 40°, 80°, 120°, and 160°. Distance in location, distance in orientation, and status of the second retrocue (stay/switch) were fully counterbalanced, resulting in 108 unique trial types. This design meant that the orientation of the two samples was the same on a fixed proportion of 1/6th of trials and the location of the two samples the same on a fixed proportion of 1/9th of trials. Responses were collected with an MRI-compatible button box.

Subjects were scanned while performing working memory for locations in a DSR task (Fig. 1). Each trial began with the 2 s presentation of two sample stimuli, followed by an initial delay period of 8 s (Delay 1.1). At 10 s, a retrocue presented (for 2 s) the orientation of the item whose location would be probed at the end of an additional 8-second-long delay period (Delay 1.2). In order to avoid differential sensory presentation of the two orientations, the retrocue contained two bars, one red and one blue, that correspond to the orientation of the two samples, and one of the colors (counterbalanced across subjects) was designated the valid cue. Subjects were told the color of the valid cue at the beginning of the experiment. The recognition probe was a white disk (radius of 2.5°) presented for 2.5 s at a location that matched the location of the cued item on 50% of trials and at a location with varying distances from that of the cued item on nonmatching trials (15°, 25°, or 35°, counterbalanced across trials). Subjects determined whether the location of the probe matched or did not match the location of the cued sample item by pressing corresponding buttons on the button box. Probe offset was followed by a 1 s unfilled delay (Delay 2.1), a second retrocue (2 s), two additional seconds of delay (Delay 2.2), and a second memory probe. The second retrocue was identical to the first, unpredictably, on 50% of trials (“stay” trials) and cued the previously uncued item on 50% of trials (“switch” trials). A white fixation dot was present throughout the trial except when replaced by the retrocues. The intertrial interval varied randomly between 6, 8, and 10 s.

Over the course of two scanning sessions, subjects performed a total of 324 trials (3 of each unique type). The first scanning session consisted of 13 runs during each of which a block of 12 trials was performed, and the second scanning session consisted of 14 run/blocks. Each run/block lasted 464 s. Before the first scanning session, each subject completed two blocks of practice trials (12 trials per block) outside of the scanner and another block of practice within the scanner before fMRI scanning began. During the fMRI scans, we tracked subjects’ eye position using an Avotec RE-5700 eye-tracking system, to monitor central fixation.

### Behavioral data analysis

We first derived a descriptive measure of each subject's performance during Probe 1 and Probe 2 by calculating the percentage of correct responses and the average response time. We compared the accuracy between Probe 1 and Probe 2 and compared the performance for *stay* and *switch* trials.

### fMRI data acquisition

Whole-brain images were acquired with a 3 T MRI scanner (Discovery MR750; GE Healthcare) at the University of Wisconsin–Madison. A high-resolution T1-weighted image was acquired with a fast-spoiled gradient-recalled echo sequence [repetition time (TR) of 8.2 ms; echo time (TE) of 3.2 ms; flip angle of 12°; 176 axial slices, 256 × 256 in-plane, 1.0 mm isotropic]. A T2*-weighted gradient echo pulse sequence was used to acquire the functional data while subjects performed the DSR task (TR of 2 s; TE of 25 ms; flip angle of 60°; 64 × 64 matrix size, 42 sagittal slices, 3 mm isotropic).

### fMRI preprocessing

Data were preprocessed using the Analysis of Functional Neuroimages (AFNI) software package (https://afni.nimh.nih.gov). Before statistical analysis, the data were first registered to the final volume of each scan with rigid-body transformations and then to the anatomical images of the first scan session, after removing the first four TRs at the beginning of each run (dummy pulses to achieve a steady state of tissue magnetization before task onset). Then volumes were motion corrected with six nuisance regressions accounting for motion artifacts in six different directions. Linear, quadratic, and cubic trends were removed for each run and then the data were *z*-scored within each run.

### Region of interest (ROI) generation

As with Yu et al. (2020), we focused the analyses on two functionally defined and anatomically constrained ROIs: early visual ROI (constrained to V1 and V2 in occipital cortex) and IPS (constrained to IPS0-5). Anatomical ROIs were generated from the probabilistic atlas of Wang et al. (2015) and warped to each subject's structural scan in their native space. To identify voxels maximally engaged by the task, we fit the fMRI data to a general linear model (GLM) containing regressors for each epoch of the task—Sample (1-TR impulse), Delay 1.1 (8 s boxcar); Cue 1 (1-TR impulse), Delay 1.2 (8 s boxcar); Probe 1 (1-TR impulse), Delay 2 (1-TR impulse); and Probe 2 (1-TR impulse), each convolved with a hemodynamic response function—along with nuisance covariates for between-scan drift and head motion. Within each anatomically defined region, the functional ROIs were defined as the 500 voxels with the strongest values for the sample regressor within the early visual ROI, and the 500 voxels with the strongest values for the Delay 1.2 regressor within IPS.

### Inverted encoding model

Multivariate inverted encoding models (Brouwer and Heeger, 2009, 2011; Ester et al., 2015; Yu and Shim, 2017) were used to reconstruct the neural representation of PMIs and UMIs. The responses of each voxel can be modeled as a weighted sum of responses from a number of hypothetical tuning channels. We used nine tuning channels for location reconstruction and six channels for orientation reconstructions. The idealized tuning curve of each channel was defined as a half-wave–rectified sinusoid raised to the eighth power for location, $R=\sin^{8}\;(x)$, and to the sixth power for orientation: $R=\sin^{6}\;(x)$, where *x* is centered on the orientation or location this channel is mostly selective to and *R* is the channel response.

For the IEM, we first estimate an encoding model with a training dataset *B*_1_ (*v* voxels × *n* trials) to characterize each voxel's selectivity $\hat{W}$ (*v* voxels × *k* channels) for the feature dimension. Then we input a new dataset *B*_2_ (*v* voxels × *n* trials) with all the voxels’ responses on a single trial to the model to reconstruct a model-based representation of memorized orientation or location *C*_2_. We can first describe the data with the following equation:

$$B_{1}=WC_{1}.$$

Here *B*_1_ represents the data matrix of BOLD responses with a size of *v* × *n* for each run where *v* is the number of voxels in the ROI and *n* is the number of trials. *C*_1_ is the idealized responses of each tuning channel for each trial (*k* channels × *n* trials). *W* (*v* voxels × *k* channels) is the weight matrix that captures the selectivity of each voxel for orientations or locations.

The first step of the IEM is to train the IEM on the training dataset *B*_1_ and compute the weight matrix $\hat{W}$ that contains each voxel's selectivity at each orientation or location channel with least-squares linear regression:

$$\hat{W}=B_{1}C_{1}^{T}(C_{1}C_{1}^{T}{)}^{-1}.$$

The next step is to invert the model with the estimated weight matrix and a new test dataset *B*_2_ (*v* voxels × *n* trials) to derive the reconstructed channel responses *C*_2_ (*k* channels × *n* trials) for each trial with the following equation:

$${\hat{C}}_{2}=({\hat{W}}^{T}\hat{W}{)}^{-1}{\hat{W}}^{T}B_{2}.$$

With this procedure, we can compute a trial-by-trial reconstruction of the maintained orientation and location for PMIs and UMIs.

The IEMs were trained and tested separately for orientation and location and separately for the early visual ROI and IPS ROI. We used a leave-one-out procedure that trained the model with data from all but one run and tested the model on the one run that was left out. We repeated this process until we computed the channel responses for all the runs. The IEMs were trained and tested on the same TR. Although we examined the time courses of IEM reconstructions from task onset until the beginning of Probe 1 (0–22 s), our hypotheses about priority-based changes in the neural representations of the UMI focused on one TR: TR 10, the final TR of Delay 1.2 (based on the findings in Yu et al., 2020). For the PMI-trained IEMs, the training labels were based on orientation or location of the PMI. After generating the reconstruction for each trial, the estimated channel responses were shifted to a common center, with 0° as the test orientation channel. Overall, we generated reconstruction of PMIs and UMIs in the two ROIs based on the PMI-trained model.

### fMRI statistical analysis

We quantified the strength of the neural reconstructions by collapsing over channel responses on both sides of the target channel (0° center), averaging them, and then used linear regression to calculate the slope for the reconstruction for each subject. A positive slope was interpreted as evidence that the dimension of sample-related information in question (location or orientation) was encoded in the same format as were the data the model was trained on. A negative slope was interpreted as evidence that the dimension of sample-related information in question (location or orientation) was encoded in a format that was remapped relative to the data the model was trained on. The magnitude of the slope was interpreted as the precision of the reconstructed neural representation. For statistical testing, we used a bootstrapping method (Ester et al., 2015, 2016) in which we randomly sampled with replacement 24 reconstructions (corresponding to *N* = 24 subjects) and took the average of the resulting channel responses, repeating this process 10,000 times. This resulted in 10,000 average orientation/location reconstructions with 10,000 slopes computed for the reconstructions. For the statistical testing, we computed two-tailed *p* values as the smaller of two resultant values—the proportion of positive slopes or the proportion of negative slopes—multiplied by 2. To compare the difference between the slopes of PMI and UMI, we randomly sampled with replacement to create a sample of 24 subjects and compute the difference in slope between PMI and UMI, repeating this process 10,000 times, and then assessed significance with the same procedure as described above.

### Data and code availability

The approved Stage 1 protocol and the study data and materials are accessible at the following link: https://osf.io/rkm39/?view_only=3ed3015fa6c94053957003c35cd8f04a.

## Results

### Behavior

Recognition accuracy for Probe 1 (*M* = 67.6%; SE = 9.7%) and Probe 2 (*M* = 65.7%; SE = 9.7%) did not differ (two-tailed paired sample *t* tests: *t*_(23)_ = 0.6; *p* = 0.56), although response time (RT) to Probe 1 (*M* = 1,142 ms, SE = 96 ms) was significantly slower than that to Probe 2 (*M* = 969 ms; SE = 78 ms; *t*_(23)_ = 5.8; *p* < 0.001). When comparing Probe 2 performance for stay versus switch trials, neither accuracy nor RT showed a significant difference (accuracy: *t*_(23)_ = 0.17, *p* = 0.86; RT: *t*_(23) _= 0.09, *p* = 0.93).

### fMRI results—time courses of IEM reconstructions

Before presenting tests of the preregistered hypotheses, we first summarize the trial-averaged time series of IEM reconstructions of stimulus location and orientation, in the two ROIs of principal interest.

#### Time courses of IEM reconstruction for location (content)

**Figure 3.**
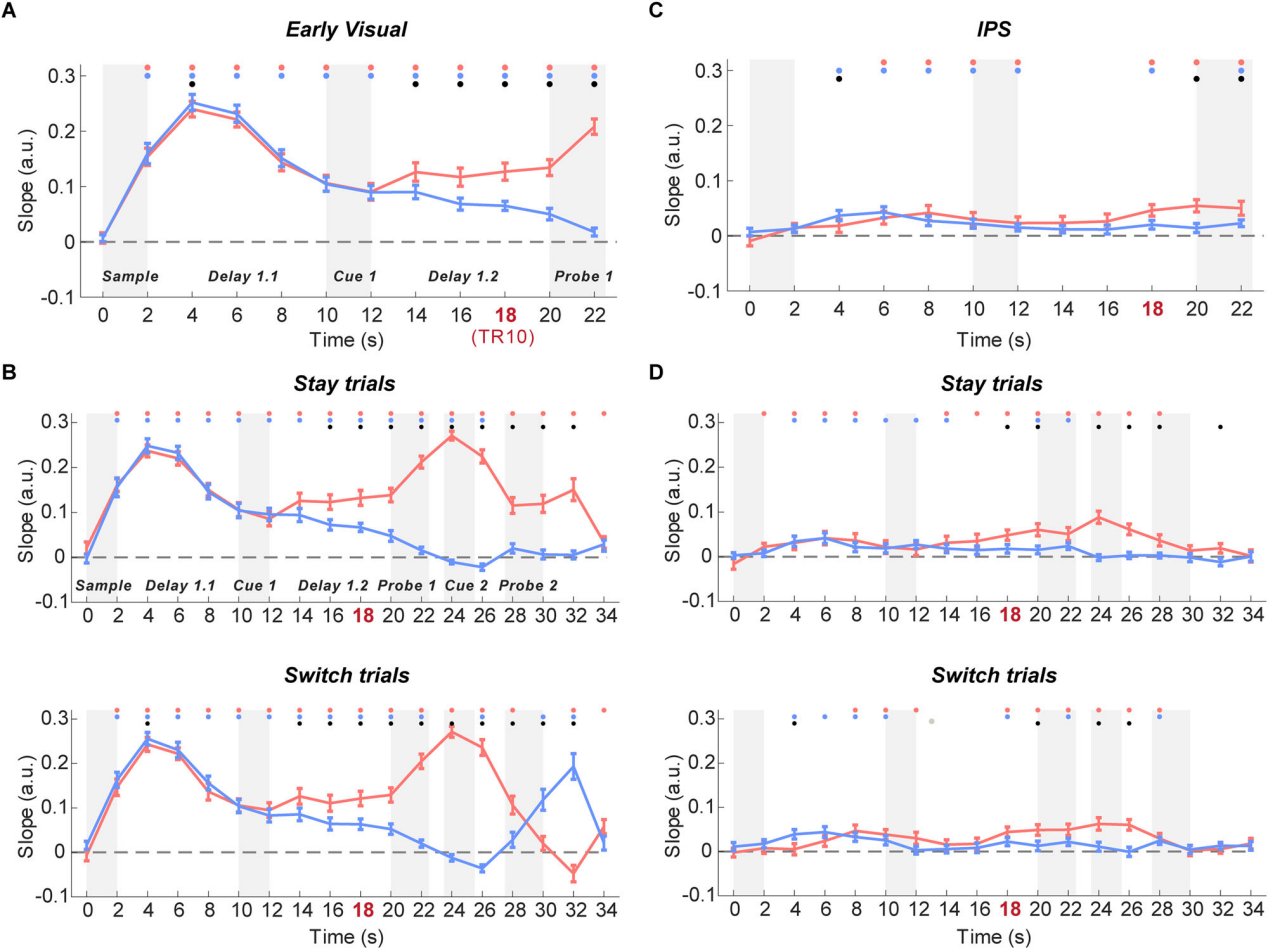
Time courses of IEM reconstructions of location content in early visual and IPS ROIs. ***A***, Time courses of the slope of location reconstructions in early visual ROI (plotted from the beginning of the trial until the end of Probe 1). ***B***, Time courses of the slope of location reconstructions throughout the whole trial in early visual ROI for stay (top) and switch (bottom) trials. ***C***, Time courses of the slope of location reconstructions in IPS ROI (plotted from the beginning of the trial until the end of Probe 1). ***D***, Time courses of the slope of location reconstructions in IPS ROI for stay (top) and switch (bottom) trials. Error bars indicate ± 1 SEM. Red, blue, and black dots indicate *p* < 0.05 for significant reconstruction of PMI, significant reconstruction of UMI, and a significant difference between the two, respectively (false discovery rate corrected across ROIs). TR 10 (18–20 s) is highlighted to show the TR of interest for the preregistered hypotheses.

In the early visual ROI, the location of both PMI and UMI could be reconstructed from Sample to Probe 1 (2–24 s; TRs 2–12; all *p*s < 0.004). Following the presentation of Cue 1, the reconstruction strengths diverged by priority status, with the representation of the location of the UMI declining monotonically, and that of the PMI significantly stronger than the UMI from 14 to 24 s (TRs 8–12; all *p*s < 0.008; Fig. 3*A*). To assess the second portion of the trial (Cue 2 to Probe 2), stay and switch trials were examined separately. On stay trials, reconstruction of the location of the PMI remained elevated until the end of the trial, whereas that of the UMI became negative from 24 to 28 s (TRs 13 and 14; *p*s < 0.013) and then no different from 0 for the remainder of the trial (*p*s > 0.12). On switch trials, the reconstruction slope of the newly cued item flipped sign from significantly negative at 26–28 s (TR 14; *p* < 0.001) to significantly positive at 30–34 s (TRs 16 and 17; *p*s < 0.001) and that of the newly uncued item flipped sign from significantly positive at 28–30 s (TR 15; *p* = 0.004) to significantly negative at 32–34 s (TR 17; *p* = 0.005; Fig. 3*B*).

In IPS, although reconstruction slopes were generally lower than for early visual cortex, the location of both PMI and UMI could be reconstructed during Delay 1.1 and presentation of Cue 1 (PMI: 6–14 s, TRs 4–7; *p*s < 0.03; UMI: 4–14 s, TRs 3–7; *p*s < 0.011). During the first portion of Delay 1.2, both dipped to baseline, with the PMI returning to positive values prior to the onset of Probe 1 (18–24 s; TRs 10–12; *p*s < 0.001), and becoming significantly different from the UMI at 20 s (TRs 11–12; *p*s < 0.031; Fig. 3*C*). Turning to the second portion of the trial, on stay trials reconstruction of the location of the PMI remained elevated from Cue 2 to Probe 2 (TRs 13–15; *p*s < 0.006) and significantly different from the UMI (TRs 13–15; *p*s < 0.006), the UMI not varying from 0. Notably, and at variance from the pattern seen in the early visual ROI, the pattern for switch trials was very similar to that from stay trials, with the slope of the newly cued item staying at baseline until briefly becoming positive from 28 to 30 s (TR 15; *p *= 0.004), and the slope of the newly uncued item staying elevated from Cue 2 to Probe 2 (TRs 13–15; *p*s < 0.012), and significantly different from the newly cued item from 24 to 28 s (TRs 13–14; *p*s < 0.004).

#### Time courses of IEM reconstruction for orientation (context)

**Figure 4.**
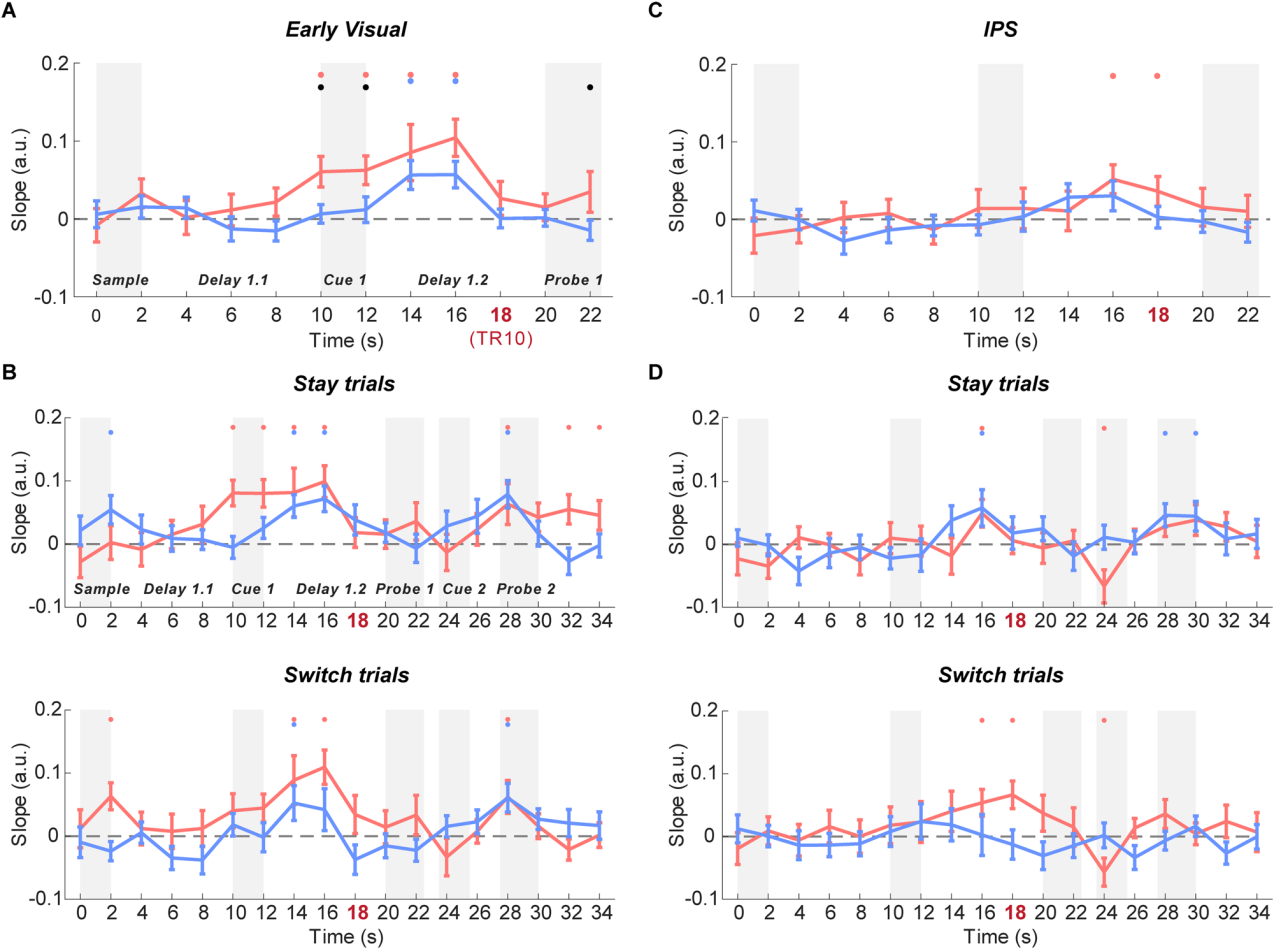
Time course of IEM reconstructions of orientation context in early visual and IPS ROIs. ***A***, Time course of the slope of orientation reconstructions in early visual ROI (from the beginning of the trial until the end of Probe 1). ***B***, Time course of the slope of orientation reconstructions for the whole trial in early visual ROI for stay (top) and switch (bottom) trials. ***C***, Time course of the slope of orientation reconstructions in IPS ROI (from the beginning of the trial until the end of Probe 1). ***D***, Time course of the slope of location reconstructions for the whole trial in IPS ROI for stay (top) and switch (bottom) trials. Red, blue, and black dots indicate *p* < 0.05 for significant reconstruction of PMI, significant reconstruction of UMI, and a significant difference between the two, respectively (false discovery rate corrected across ROIs). Error bars indicate ± 1 SEM.

In the early visual ROI, the orientation of the PMI could be reconstructed during Cue 1 and early Delay 1.2 (TRs 6–9; 10–18 s; *p*s < 0.02) and the orientation of the UMI during early Delay 1.2 (TRs 8 and 9; 14–18 s; *p*s < 0.011), which then both returned to baseline during late Delay 1.2 and Probe 1 (TRs 10–12; *p*s > 0.15; Fig. 4*A*). Turning to the second portion of the trial (Cue 2 to Probe 2), on stay trials, reconstruction of the orientation of the PMI was successful during and after Probe 2 (TRs 15, 17, and 18; *p*s < 0.043), as was the reconstruction of the UMI during Probe 2 (TR 15; *p* = 0.022; Fig. 4*B*). On switch trials, a similar pattern was observed, with the orientation of both PMI and UMI reconstructable at TR 15 (*p*s < 0.02).

In IPS, the orientation of the PMI could be reconstructed during Delay 1.2 (TRs 9 and 10; 16–20 s; *p*s < 0.04). The orientation of the UMI could not be reconstructed at any TR between Sample and Probe 1 (*p*s > 0.08). For the second portion of the trial, on stay trials the orientation of the PMI remained at baseline and briefly became negative at TR13 (24–26 s; *p *< 0.001), whereas the orientation of the UMI could be briefly reconstructed during Probe 2 (TRs 15 and 16; *p*s < 0.032). On switch trials, the slope of the PMI once again dipped below baseline at TR 13 (*p *= 0.002) and the orientation of the UMI could not be reconstructed throughout Cue 2 to Probe 2 (*p*s > 0.066).

### fMRI results—preregistered hypothesis tests

**Figure 5.**
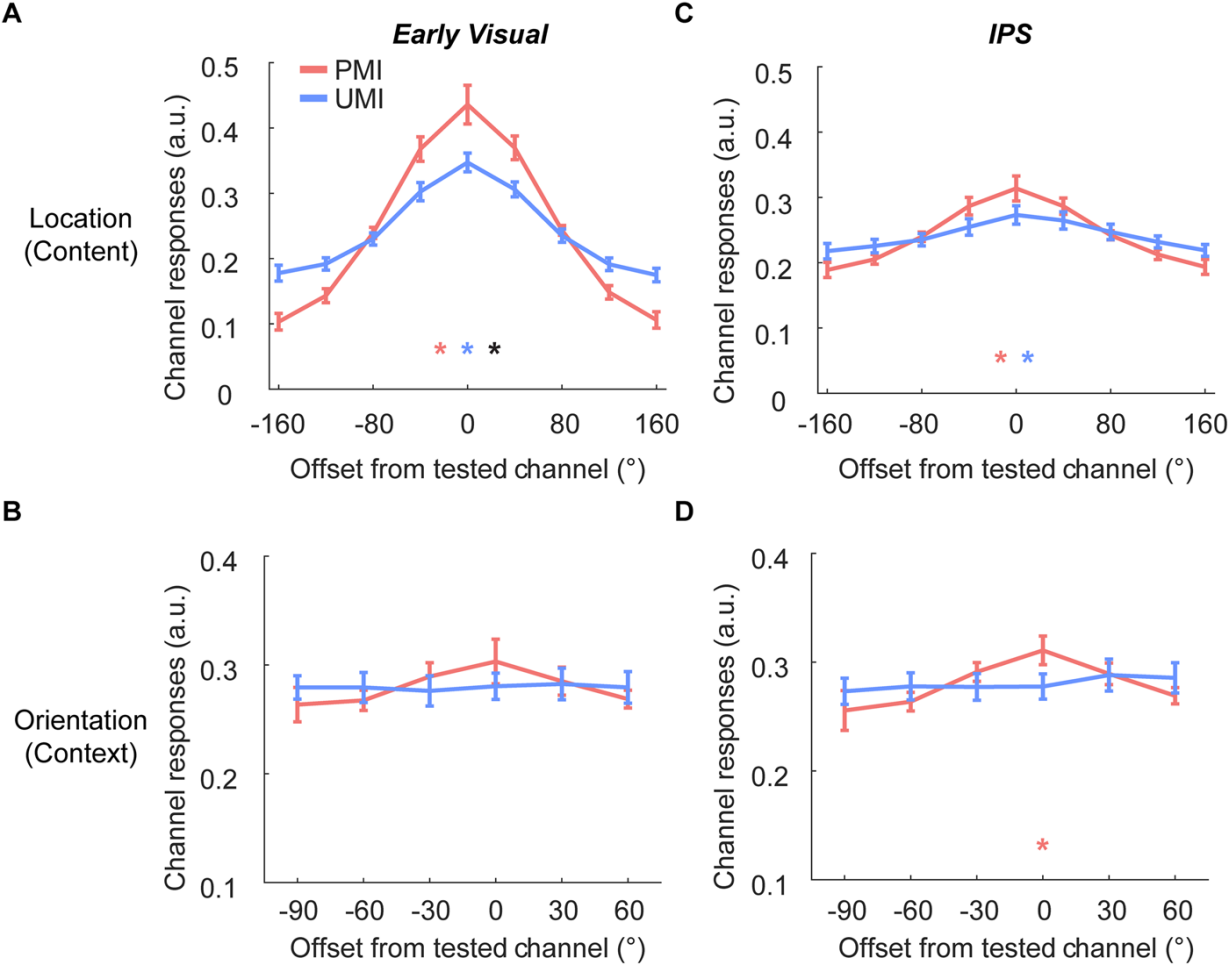
IEM reconstructions of location content and orientation context during late Delay 1.2. ***A***, IEM reconstructions of stimulus location (content) in early visual ROI at TR 10 (18 s after trial onset). ***B***, IEM reconstructions of stimulus orientation (context) in early visual cortex at TR 10. ***C***, IEM reconstructions of stimulus location in IPS at TR 10. ***D***, IEM reconstructions of stimulus orientation in IPS at TR 10. Error bars indicate ± 1 SEM. Red, blue, and black dots indicate *p* < 0.05 for significant reconstruction of PMI, significant reconstruction of UMI, and a significant difference between the two, respectively (all *p* values are false discovery rate corrected).

The main hypotheses focused on the IEM reconstructions at TR 10 (18–20 s after trial onset), which corresponds to late Delay 1.2, and should be uncontaminated by Probe 1-related activity. Results plotted in Figure 5 use the same graphical conventions as Figure 2's diagrams of idealized predictions of the three models, to facilitate comparison.

#### Content of PMI in early visual cortex

The reconstruction of the location content of the PMI with a PMI-trained model had a significant positive slope (*p* < 0.001; Fig. 5*A*). (This result was consistent with the predictions of all three models.)

#### Context of PMI in early visual cortex

The reconstruction of the orientation of the PMI with a PMI-trained model did not differ from 0 (*p* = 0.11; Fig. 5*C*). (This result was consistent with the *functional model* and inconsistent with the other two.)

#### Content of UMI in early visual cortex

Cross-decoding of the location of the UMI with a PMI-trained model produced a significant positive slope (*p* < 0.001; Fig. 5*A*). (This result was not consistent with any of the models.)

#### Context of UMI in early visual cortex

Cross-decoding of the orientation of the UMI with a PMI-trained model produced a nonsignificant reconstruction (*p* = 0.78; Fig. 5*C.*). (This result was consistent with the *functional model* and inconsistent with the other two.)

#### Content of PMI in IPS

The reconstruction of the location content of the PMI with a PMI-trained model showed a significantly positive slope (*p* = 0.0013; Fig. 5*B*). (This result was consistent with the *domain-dependent* and *hybrid* models and inconsistent with the *functional model*.)

#### Context of PMI in IPS

The reconstruction of the orientation context of the PMI with a PMI-trained model showed a significantly positive slope (*p* = 0.04; Fig. 5*D*). (This result was consistent with the *functional* and *hybrid* models and inconsistent with the *domain-dependent* model.)

#### Content of UMI in IPS

Cross-decoding of the location of the UMI with a PMI-trained model produced a significantly positive slope (*p* = 0.004; Fig. 5*B*). (This result was inconsistent with all the models.)

#### Context of UMI IPS

Cross-decoding of the orientation of the UMI with a PMI-trained model produced a nonsignificant reconstruction (*p* = 0.78; Fig. 5*D*). (This result was consistent with the *domain-dependent* model and inconsistent with the other two.)

### Interim discussion

Results from the preregistered analyses were not fully consistent with any of the three a priori-defined models. When considering early visual cortex alone, however, the results were most conceptually consistent with the functional account, because in this region the feature acting as content (location) was sensitive to priority, but the feature acting as context (orientation) was not. Indeed, we failed to find any evidence in early visual cortex for the active representation of the feature acting as context, a fact that we will return to in the Discussion. For IPS, the results were most consistent with the hybrid model: The feature acting as context (orientation) could be reconstructed in IPS, but so too could location, a stimulus domain that, despite acting as content in this study, may be fundamental to the representational functions of this region. A complication for both of these interpretations, however, is the failure to observe negative reconstructions of the UMI, in either region and for either stimulus feature, during late Delay 1.2. We will also consider this in more detail in the Discussion. (Note that we performed additional analyses excluding trials where the two samples had identical orientation or location, and the results revealed qualitatively similar patterns to those reported.)

Due in part to the equivocal nature of the results of the preregistered analyses, we subsequently carried out a series of exploratory analyses that, although not part of the preregistration, may generate results that can inform our understanding of the role of IPS in representing the priority status of content and context in working memory.

### Exploratory analyses

**Figure 6.**
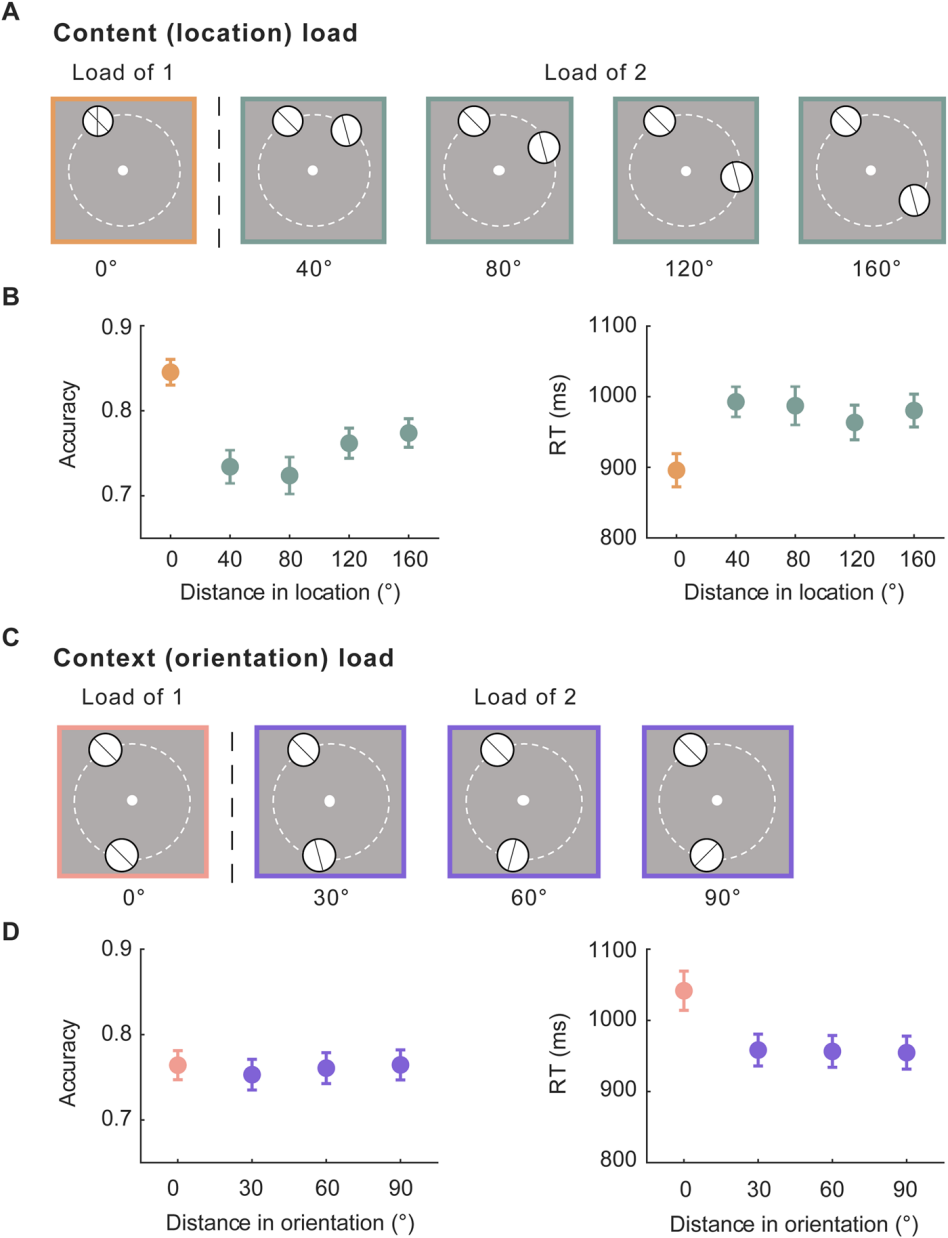
Load-dependent sensitivity of recognition performance. ***A***, Depiction of content (location) load. ***B***, Accuracy (left) and RT (right) plotted as a function of the distance in location between the two stimuli. ***C***, Depiction of context (orientation) load. ***D***, Accuracy (left) and RT (right) plotted as a function of the distance in orientation between the two stimuli. Error bars indicate ± 1 SEM.

When considering working memory functions of IPS, two important characteristics are that univariate activity in this region is sensitive to working memory load (Todd and Marois, 2004, 2005; Xu and Chun, 2006) and to context-binding demand (Gosseries et al., 2018; Cai et al., 2022; Fulvio et al., 2023). Building from this, we designed these exploratory analyses with the goal of disentangling effects of the memory load of stimulus content from the effects of the memory load of stimulus context. In our DSR task, content load varied with the distance (in location on the screen) between the two stimulus items, with a content-load-of-1 when the two items shared the same location and a content-load-of-2 when they did not. Furthermore, difficulty due to inter-item interference could also be assumed to vary within content-load-of-2 displays (Fig. 6*A*). The same was true for context load, when considering inter-item distance in orientation (Fig. 6*C*). Using these properties of our design, the first exploratory analysis tested load-dependent changes in the univariate BOLD activity in early visual cortex and in IPS, for content (location) load and for context (orientation) load. For a second exploratory analysis, we adopted an individual-differences approach to assess the correlation between individual differences in load-dependent modulation on BOLD activity and individual differences in behavioral signatures of load modulation.

#### Exploratory analyses: methods and predictions

##### fMRI preprocessing

The data were preprocessed following the same procedures as described in the preregistered methods, with the exception that data were spatially smoothed with a Gaussian kernel with 4 mm full-width at half-maximum. Voxel selection from the two ROIs followed the same procedures as described in the preregistered methods. Percentage signal change was calculated by baseline-correcting the BOLD signal intensity to the first TR of each trial.

##### Inferential statistics assessing load sensitivity

Because the locations and orientations of the two samples were selected independently, each trial had one of five levels of distance in location (polar angle), 0°, 40°, 80°, 120°, and 160°, and one of four levels of distances in orientation: 0°, 30°, 60°, and 90°.

For the behavioral data, accuracy for Probe 1 and RT for Probe 1 were evaluated with repeated-measures one-way ANOVAs that treated distance in location as a factor with five levels and distance in orientation as a factor with four levels. Where appropriate, follow-up pairwise tests assessed whether load-of-1 trials (i.e., 0°) may have been categorically different from load-of-2 trials. fMRI data were trial-averaged by stimulus distance, separately for content and for context, and differences at each time point were assessed by repeated-measures one-way ANOVAs with five levels for content and four levels for context.

Based on the behavioral results, which did show evidence for categorical differences between load-of-1 trials and load-of-2 trials, the trial-averaged BOLD time courses were assessed for differences between load-of-1 trials and load-of-2 trials, at each time point, with permutation tests with 10,000 iterations.

For IPS, the three models made different predictions for activity during Delay 1.2: the *domain-dependent* model predicted that this activity would be sensitive to content (location) load, but not context (orientation) load; the *functional* model predicted that this activity would not be sensitive to content load, but that it would be sensitive to context load; and the *hybrid* model predicted that it would be sensitive to variation of load along both dimensions. For early visual cortex, however, based on previous results (Gosseries et al., 2018; Cai et al., 2022; Fulvio et al., 2023), we did not expect to find evidence for delay-period load sensitivity.

##### Relating load sensitivity in behavior to load sensitivity in delay-period activity

At the individual subject level, behavioral load sensitivity was calculated by subtracting the average RT for load-of-1 trials from the average RT for load-of-2 trials, separately for content load and context load, and neural load sensitivity in IPS, during Delay 1.2 (TRs 8–10), was calculated by subtracting the average BOLD signal intensity from for load-of-1 trials from the average activity for load-of-2 trials, separately for content and context load. With these values, Pearson’s correlation coefficients were calculated to assess the relationship of individual differences in behavioral load sensitivity with individual differences in neural load sensitivity. For completeness, we repeated these analyses with measures of neural load sensitivity from early visual cortex.

Once again, for IPS the three models made different sets of predictions: the *domain-dependent* model predicted a significant correlation of behavioral content-load sensitivity with neural content-load sensitivity, but no such correlation for context-load sensitivity; the *Functional* model predicted no correlation of behavioral content-load sensitivity with neural content-load sensitivity, but a significant correlation for context-load sensitivity; and the *hybrid* model predicted significant behavior–brain correlations for both content-load sensitivity and context-load sensitivity.

#### Exploratory analyses: results

##### Behavioral sensitivity to memory load

Beginning with stimulus content (location), accuracy was markedly higher, and RT lower, for 0° trials than those for the other distances (Fig. 6*B*). For accuracy, ANOVA revealed a significant main effect of stimulus distance (*F*_(4,92)_ = 20.2; *p* < 0.001), with follow-up pairwise comparisons confirming that accuracy for 0° trials was higher than that for the other four distances (all *t*s > 4.8; *p* < 0.001; after Bonferroni–Holm corrections) and that accuracy on 40° and 80° trials was lower than that on the 160° condition (all *t*s > 2.6; *p*s < 0.049; after Bonferroni–Holm corrections; other comparisons n.s.). For RT, ANOVA also revealed a significant main effect of location distance (*F*_(4,92)_ = 10.3; *p* < 0.001), the follow-up pairwise comparison confirming that responses on 0° trials were faster than those on the other three distances (all *t*s > 3.9; *p* < 0.001; after Bonferroni–Holm corrections; other comparisons n.s.). For stimulus context (orientation), accuracy did not appear to vary with load, but RT was markedly slower for 0° trials than that for the other distances (Fig. 6*D*). For accuracy the repeated-measures ANOVA confirmed that there was no main effect (*F*_(3,69)_ = 0.3, *p* = 0.8). For RT there was a significant main effect of distance (*F*_(3,69)_ = 14.8; *p* < 0.001), with follow-up pairwise comparisons confirming that responses on 0° trials were slower than those on the other three distances (all *t*s > 5.3; *p* < 0.001; after Bonferroni–Holm corrections; other comparisons n.s.).

##### Neural sensitivity to memory load

**Figure 7.**
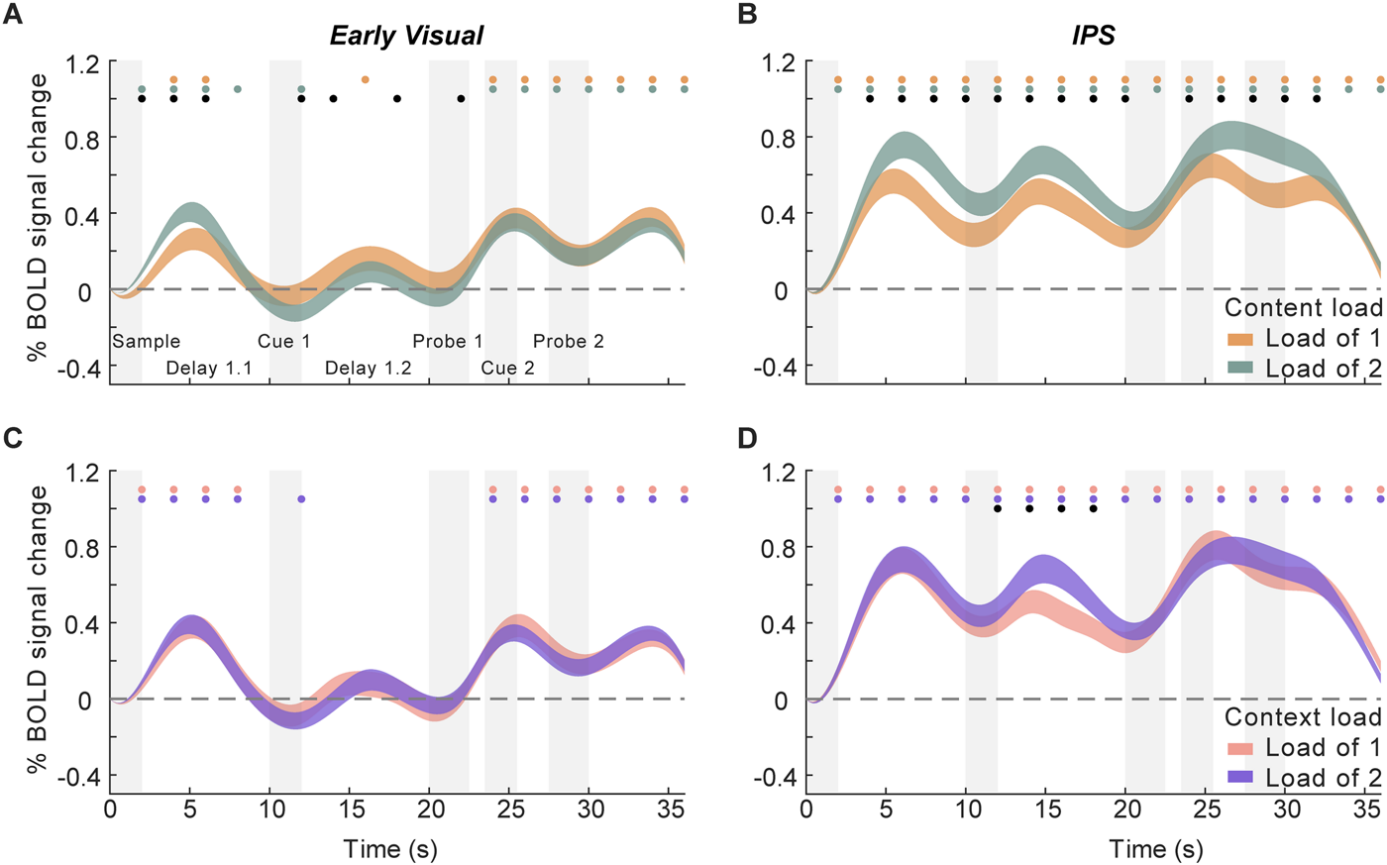
Load sensitivity in trial-averaged fMRI activity. ***A***, Activity in early visual cortex, sorted by content (location) load. Orange, green, and black dots indicate *p* < 0.05 for differences for load-of-1 trials versus baseline, load-of-2 trials versus baseline, and between the two trial types, respectively (false discovery rate corrected). ***B***, Activity in IPS, sorted by content (location) load (conventions same as ***A***). ***C***, Activity in early visual cortex, sorted by context (orientation) load. Pink, purple, and black dots indicate *p* < 0.05 for differences for load-of-1 trials versus baseline, load-of-2 trials versus baseline, and between the two trial types, respectively (false discovery rate corrected). ***D***, Activity in IPS, sorted by context (orientation) load (conventions same as ***C***). Extended Data Figure 7-1 shows load sensitivity in trial-averaged fMRI activity sorted by distance in location (Fig. 7-1A, 7-1B) or orientation (Fig. 7-1C, 7-1D) between the two memory items.

For stimulus content (location), in the early visual ROI the initial sample-evoked response was greater for load-of-2 than on load-of-1 trials (2–8 s after trial onset; TRs 2–4; *p*s < 0.001), after which activity in both conditions returned to baseline and fluctuated near that level across Cue 1 and Delay 1.2. [See Fig. 7*A* for individual time points where load-of-1 activity differed from load-of-2 (but not baseline) or where it differed from baseline (but not load-of-2; *p*s < 0.015).] Activity for both types of trial then increased comparably in response to Probe 1 and remained elevated for the remainder of the trial. In IPS, activity for both trial types was elevated throughout the trial and with the exception of the very beginning and end was significantly greater for load-of-2 than that for load-of-1 throughout (*p*s < 0.022) with the exception of one time point (TR 12; *p* = 0.32).

10.1523/ENEURO.0474-23.2024.f7-1 Figure 7-1 **Load-sensitivity in trial-averaged fMRI activity.** (A) Activity in early visual cortex, sorted by distance in location between the two memory items (5 levels). Black dots indicate p < 0.05 for the main effect of location distance from repeated measures one-way ANOVAs (false discovery rate corrected). (B) Activity in IPS, sorted by distance in location (conventions same as A). (C) Activity in early visual cortex, sorted by distance in orientation (4 levels). Black dots indicate p < 0.05 for the main effect of orientation distance from repeated measures one-way ANOVAs (false discovery rate corrected). (D) Activity in IPS, sorted by distance in orientation (conventions same as C). Download Figure 7-1, TIF file.

For stimulus context (orientation), the early visual ROI activity for both trial types increased for sample presentation, returned to baseline and fluctuated near that level across Cue 1 and Delay 1.2, and then increased in response to Probe 1 and remained elevated for the remainder of the trial, never differing by trial type. In IPS, activity for both trial types was elevated throughout the trial and was greater on load-of-2 than load-of-1 trials from Cue 1 through much of Delay 1.2 (12–20 s; TRs 7–10; *p*s < 0.013). [Repeated-measures one-way ANOVAs on activity sorted by location and orientation distance confirmed the main effects of location and orientation distances and suggested a similar categorical effect (load-of-1 vs load-of-2) as the behavioral effect.]

The results from this set of exploratory analyses were consistent with the *hybrid* model.

Additionally, it is important to note that the load-related effects on IPS activity can not be solely attributed to task difficulty. Although IPS activity did increase with task difficulty for content (location) load, as indicated by higher RT on content load-of-2 trials, it decreased with task difficulty for context (orientation) load, as evidenced by higher RT on context load-of-1 trials.

##### Relating load sensitivity in behavior to load sensitivity in delay-period activity

**Figure 8.**
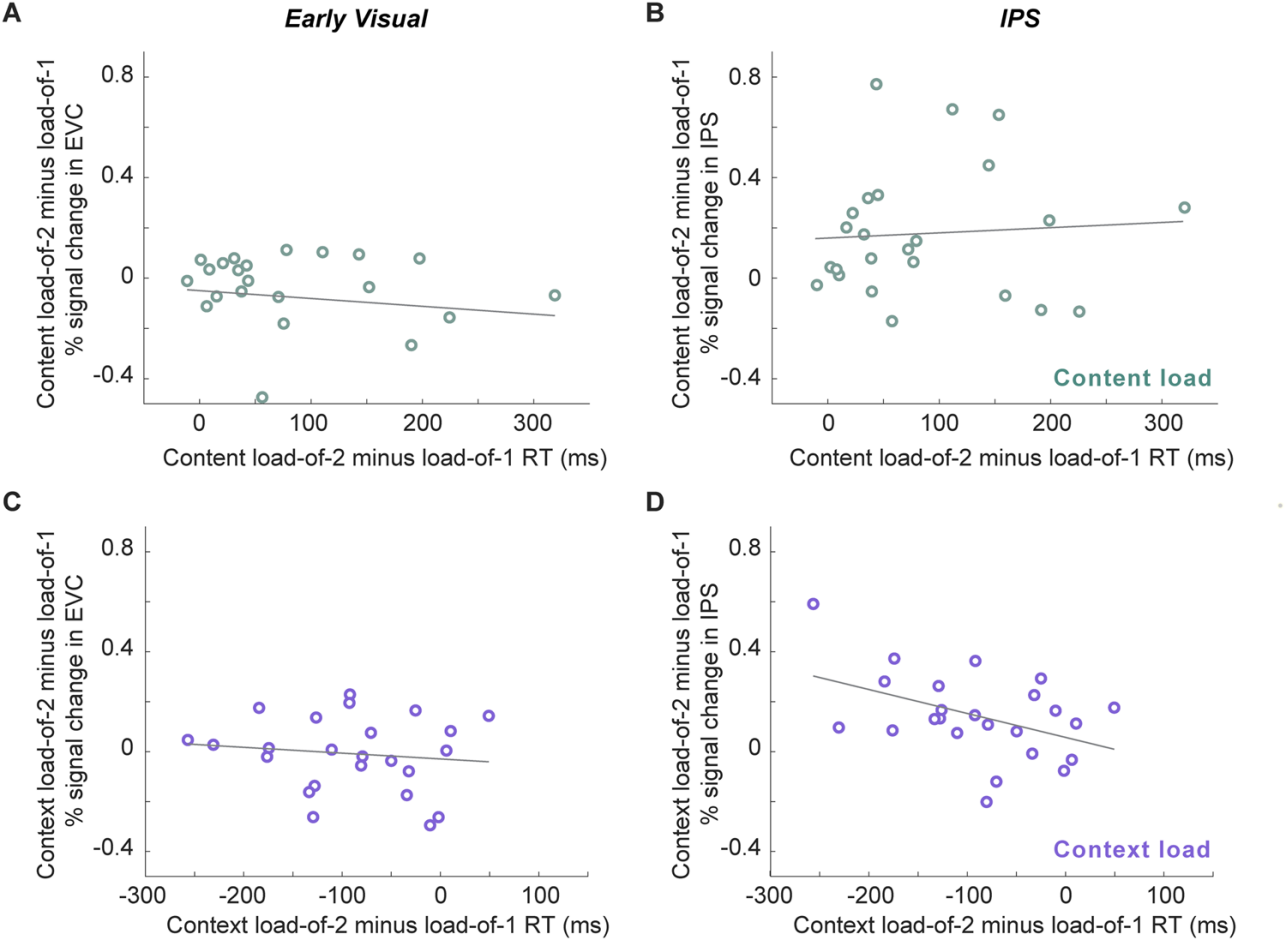
Relating load sensitivity in behavior to load sensitivity in delay-period activity. ***A***, For each individual, we quantified content-load modulation of early visual ROI activity by subtracting content-load-of-2 activity by content-load-of-1 activity during Delay 1.2 and correlating it with each individual's content-load modulation on RT, which turned out to be nonsignificant (*r*^2^ = 0.014; *p* = 0.58). ***B***, Correlation between individual differences in content-load modulation of IPS activity and individual differences in content-load modulation of RT was not significant (*r*^2^ = 0.012; *p* = 0.62). ***C***, Correlation between context-load modulation of early visual ROI activity and context-load modulation of RT was not significant (*r*^2^ = 0.008; *p* = 0.67). ***D***, A significant negative correlation was found between context-load modulation of IPS activity and context-load modulation of RT (*r*^2^ = 0.19; *p* = 0.03).

Beginning with content (location) load, for IPS individual differences in behavioral load sensitivity did not correlate with individual differences in neural load sensitivity (*r*^2^ = 0.012; *p* = 0.62; Fig. 8*B*). Similarly, for early visual cortex, individual differences in behavioral load sensitivity did not correlate with individual differences in neural load sensitivity (*r*^2^ = 0.014; *p* = 0.58; Fig. 8*A*).

Turning to context (orientation) load, in IPS, individual differences in behavioral load sensitivity were significantly correlated with individual differences in neural load sensitivity (*r*^2^ = 0.19; *p* = 0.03), reflecting the fact that greater load-related increases of BOLD activity were associated with greater load-related slowing in RT (Fig. 8*D*). For early visual cortex, individual differences in behavioral load sensitivity did not correlate with individual differences in neural load sensitivity (*r*^2^ = 0.008; *p* = 0.67; Fig. 8*C*).

The results from this final set of exploratory analyses were consistent with the *functional* model.

## Discussion

The flexible, context-dependent control of thought and behavior depends on the flexible, context-dependent control of the contents of working memory (WM). This has previously been studied with DSR of a memory set of two simultaneously presented orientation patches, with the results indicating that different brain areas are important for representing the prioritization of different dimensions of stimulus information: The priority-based transformation of stimulus orientation was only observed in early visual cortex, whereas the priority-based transformation of stimulus location was only observed in IPS (Yu et al., 2020). What these results left unresolved, however, was whether this dissociation reflected the relative specialization of these two regions for the representation of orientation versus of location, respectively, or alternatively, if it reflected differential roles in the processing of to-be-remembered content versus the context that was used to cue priority. This preregistered study sought to resolve this ambiguity by presenting subjects with a stimulus array identical to the earlier study but reversing the roles of orientation versus location assigned to the two critical stimulus dimensions. Although the results did not conform to all of the predictions made by any one of the three a priori-defined models, on balance they were consistent with a *functional* account of WM activity in early visual cortex and a *hybrid* account of WM activity in IPS.

Perhaps the most striking aspect of the present results was the failure to find strong evidence for the active delay-period representation of stimulus orientation in early visual cortex: It was absent during Delay 1.1, and the significant IEM reconstructions observed during and immediately following the presentation of Cue 1 were likely due, at least in part, to the fact that Cue 1 visually re-presented these two orientations. Initially, this might seem to be at odds with one of the most replicated findings in visual WM research, one that dates back to some of the earliest applications of multivariate decoding methods to fMRI data (Harrison and Tong, 2009; Serences et al., 2009). But this result, together with the robust and priority-sensitive representation of stimulus location, was predicted by the *functional* model. Thus, delay-period stimulus representation that is commonly observed in early visual cortex may be due primarily to the fact that the information being decoded is the to-be-remembered content. Consistent with this view is work suggesting a necessary role for early visual cortex in maintaining stimulus content in WM (Hallenbeck et al., 2021).

In IPS we observed a pattern most consistent with the *hybrid* model, for which the preregistered prediction was active representation, during late Delay 1.2, of the orientation of the prioritized item (serving as context), as well as of the location of both items. A role for IPS in the representation of stimulus location is well established (Itti and Koch, 2001; Serences and Yantis, 2007; Jerde et al., 2012; Sprague and Serences, 2013). Further consistent with an important role in the processing of context was the evidence, from an exploratory analysis, that the sensitivity of IPS to context load predicted behavior sensitivity to this factor. The present findings thus add to a growing body of evidence that delay-period activity of IPS plays an important role in the binding of context to content (Gosseries et al., 2018; Cai et al., 2022) and that the representation of stimulus context in IPS is sensitive to task demands (Fulvio et al., 2023). Tracking context, including for nonspatial features (Greenberg et al., 2010; Liu et al., 2011; Gosseries et al., 2018; Sheldon et al., 2021), is a property consistent with the function of a frontoparietal priority map (Bisley and Mirpour, 2019).

An important question for WM research is understanding why seemingly redundant representations of stimulus information can be decoded from multiple brain regions, including in early visual, parietal, and frontal cortex (Lorenc et al., 2018; Cai et al., 2019; Rademaker et al., 2019; Hallenbeck et al., 2021). One proposition is that representations in IPS are resistant to being “overwritten” by subsequently processed visual information and so may be important for distractor resistance (Bettencourt and Xu, 2016; Lorenc et al., 2018; Rademaker et al., 2019). For example, in a task in which orientation was the to-be-remembered content, representations in IPS were found to be unbiased by distraction and recoded into a nonsensory code (Rademaker et al., 2019). The same may not be true, however, if location is the to-be-remembered content of WM (Hallenbeck et al., 2021). Of possible relevance to the “multiple copies” question is the difference that we observed in the representation of location in early visual cortex versus in IPS—only the former was sensitive to priority status. Furthermore, although delay-period activity of IPS was sensitive to variation of content load, this did not covary with behavioral sensitivity to the same factor. One implication of these findings, and, more broadly, of the *functional* model that we tested here, is that stimulus representations in early visual cortex may be of particular importance for guiding responses based on the contents of visual WM. This is also consistent with the proposal that representations in the early visual cortex have higher fidelity than representations in more anterior regions (Christophel et al., 2018).

One aspect of our results that was not predicted was that during late Delay 1.2, we did not observe significantly negative IEM reconstructions of the UMI for either location content (expected for early visual cortex by the *functional* model) or orientation context (expected for IPS by both the *functional* and *hybrid* models). (Negative reconstructions of the location of the UMI were observed in early visual cortex later in the trial, during both stay and switch trials.) This raises questions about the boundary conditions of when such representational transformation can be observed in patterns of activity. We note that work carried out after the preregistration of this study has shown that priority-based representational transformations occur in low-dimensional subspaces in recurrent neural network (RNN) simulations of WM (Wan et al., 2022) and that this has also been observed in an EEG dataset that did not show negative decoding (Wan et al., unpublished). Understanding the conditions under which priority-based transformations are, versus are not, observable in multivariate decoding analyses is therefore an important question for future research.

Work that postdated the preregistration of this study has indicated that prioritization may also be reflected in representational dynamics within low-dimensional subspaces, dynamics that would not be detectable by the analysis methods used here. An important question for future work is understanding how multivariate decoding methods respond to the dynamics of representational subspaces.

To conclude, this registered report was designed to differentiate the roles of early visual cortex and IPS in representing the priority of stimulus content versus of stimulus context in visual WM. We found that early visual cortex encoded the representations of location content in a priority-sensitive manner and failed to find evidence in this region for the active representation of orientation when this stimulus feature had the role of context in the DSR task. IPS also encoded stimulus location, although not in a manner that was sensitive to priority. IPS also encoded the representation of prioritized orientation context and did so in a manner that predicted behavioral sensitivity to context load. These results highlight the task-dependent coding of WM representations, such that for a given piece of information, where and how it is processed in WM depends on its role in the task that's being carried out.

## Synthesis

Reviewing Editor: Frederike Beyer, Queen Mary University of London Decisions are customarily a result of the Reviewing Editor and the peer reviewers coming together and discussing their recommendations until a consensus is reached. When revisions are invited, a fact-based synthesis statement explaining their decision and outlining what is needed to prepare a revision will be listed below. The following reviewer(s) agreed to reveal their identity: Xuqian Li.

Apologies for the delay in coming to a decision regarding your manuscript, I've been waiting for a second review to be submitted. In order to not further delay the processing of your manuscript, I have now decided to proceed with the review we have received. As you can see in the reviewer's comments below, they are satisfied that the main hypotheses have been addressed adequately in the analyses, but raise some issues with the exploratory analyses. Please ensure these points are addressed in the revision.

Best wishes,

Frederike Beyer

Reviewer comments

1. Whether the data are able to test the authors' proposed hypotheses by passing the approved outcome-neutral criteria (such as absence of floor and ceiling effects or success of positive controls)?

Yes. The data in general are suitable for testing the domain-dependent, functional, and hybrid models. The time courses of IEM reconstruction of stimulus location (Figure 3), as measured by the slope of the tuning function, clearly showed that throughout Delay 1.1, where both samples were just presented and no priority was ascribed to any one sample, the reconstruction of both samples were significant regardless of the trial types. These observations are consistent with expectations and early reports, which suggest the adequacy of the acquired dataset and analyses to test the critical hypotheses in relation to Delay 1.2.

2. Whether the Introduction, rationale and stated hypotheses are the same as the approved Stage 1 submission?

Yes. The introduction, rationale, and stated hypotheses are the same as the Stage 1 submission.

3. Whether the authors adhered to the registered experimental procedures?

Yes. The authors adhered to the registered dual-serial retro-cueing experiment.

4. Whether any unregistered exploratory analyses are justified, methodologically sound, and informative?

The authors performed exploratory analyses to test (a) how memory load of content and context affected averaged BOLD responses, and (b) whether the load-related BOLD activity was related to the load-related modulations in behavioural performance. My major concern is about the load-of-1 trials. For location (content), a load-of-1 situation (as shown in Figure 6a) shows no need to memorise the orientation (context) at all, because people knew exactly which location would be tested at Probe 1 and Probe 2, which is much easier than the load-of-2 situation. Hence, when comparing load-1 and load-2 content, the results could be confounded with task difficulty. For orientation (context), a load-of-1 situation (as shown in Figure 6c) shows no way to know which one of the locations (content) is cued because the cues showing as lines of different colours are overlapped. In this case, participants are not benefited from the cue that is supposed to allow people to only focus on one sample. What's even worse is that participants may not be able to know which one of locations is tested at Probe 1 and Probe 2, which makes the task very difficult. This idea can be traced by the exploratory behavioural result showing that the RT for load-1 trials is slower than load-2 trials (Figure 6D). Hence, the results of the load effect of context could be confounded with task difficulty.

While here I mainly focus on the load-1 versus load-2 comparison here, I would also like to encourage the authors to perform additional IEM analyses without the load-1 trials, because either load-1 of content or context would make the priority manipulation invalid at Delay 1.2, which inevitably adds noises to feature reconstruction.

5. Whether the authors' conclusions are justified given the data?

Yes. In general, the conclusions can be justified given the data. However, I would recommend the authors to be cautious about making claim like "...and did so in a manner that predicted behavioural sensitivity to context load" since that the load-dependent exploratory results can be confounded with task difficulty.

6. Minor point. Please clarify the response participants need to make at Probe 1 and Probe 2 (e.g., decide whether the probe item match or mismatch the sample item).
