# Supplementary figures and images for: Investigating the Roles of the Visual and Parietal Cortex in Representing Content versus Context in Visual Working Memory
Source: eNeuro. 2024 Feb 6;11(2):ENEURO.0270-20.2024. doi: 10.1523/ENEURO.0270-20.2024 (PMC10860598; doi:10.1523/ENEURO.0270-20.2024)

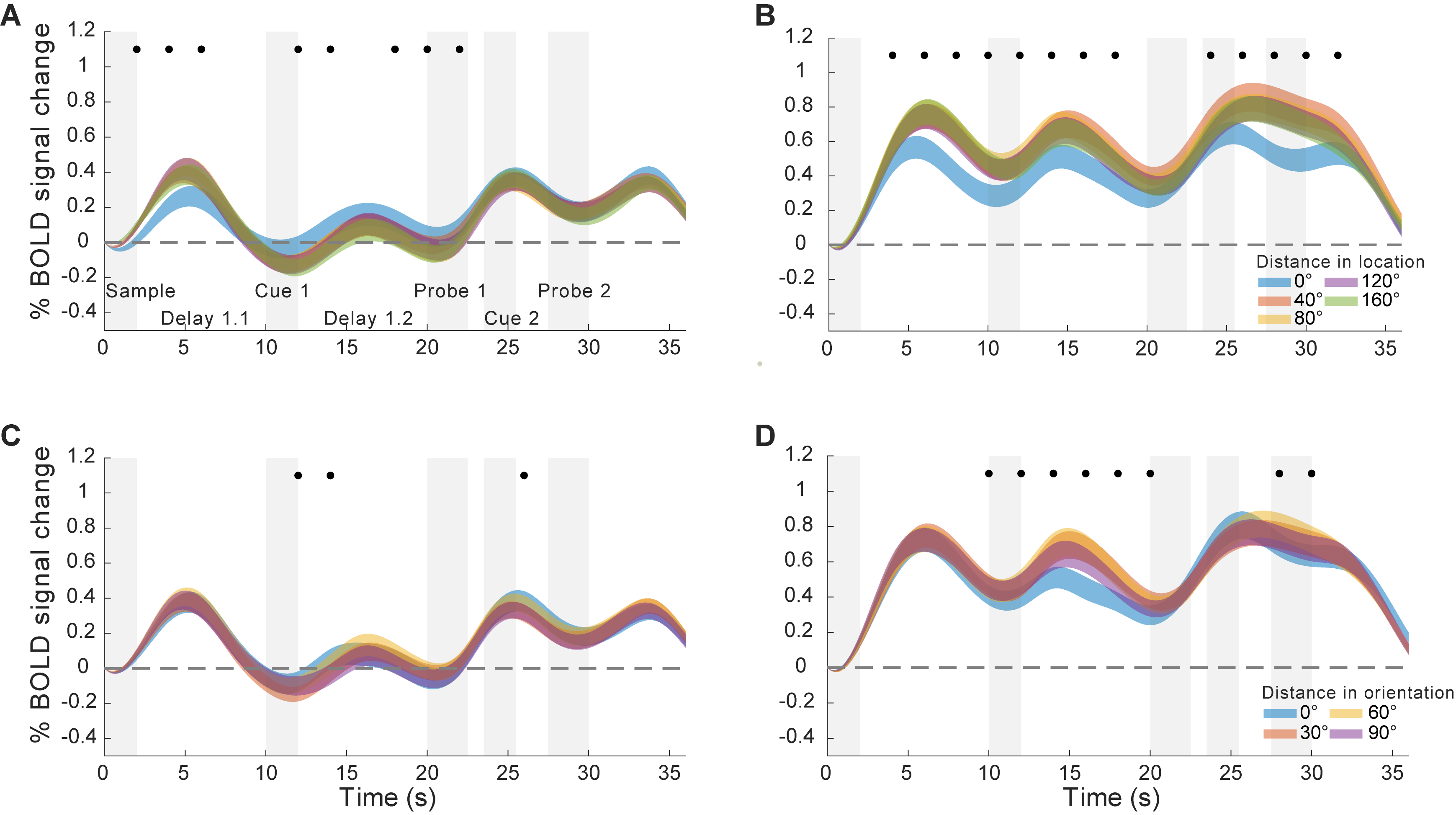

Supplement: Figure 7-1 — Load-sensitivity in trial-averaged fMRI activity. (A) Activity in early visual cortex, sorted by distance in location between the two memory items (5 levels). Black dots indicate p < 0.05 for the main effect of location distance from repeated measures one-way ANOVAs (false discovery rate corrected). (B) Activity in IPS, sorted by distance in location (conventions same as A). (C) Activity in early visual cortex, sorted by distance in orientation (4 levels). Black dots indicate p < 0.05 for the main effect of orientation distance from repeated measures one-way ANOVAs (false discovery rate corrected). (D) Activity in IPS, sorted by distance in orientation (conventions same as C). Download Figure 7-1, TIF file. [file eneuro-11-ENEURO.0270-20.2024-s002.tif]
